# Trivialities

**Published:** 1890-08-02

**Authors:** 


### Passing Topics.

### TRIVIALITIES.

It is to be regretted that the inquiry by the Lords' Select Committee, which promised to be thorough, now threatens to be diffuse, and it seems not unlikely that the mind of the Committee will be filled to repletion with a heap of petty details and exercised to exhaustion with a variety of subordinate issues to the exclusion and neglect of those momentous questions which it was hoped would be brought nearer to elucidation and settlement by their lordships' aid.

We can conceive few things more lamentable than the exhibition of personalities which it has pleased certain witnesses to parade, and if it be true that anything in the nature of collusion has occurred—a possibility openly discussed—the Committee will no doubt find means to mark their sense of the attempt to prostitute the proceedings to the purposes of an ignoble feud.

Day after day, for a considerable period, the evidence seems little more or less than a series of attacks upon the London Hospital, interspersed by the defence, and while we are specially desirous to avoid anything which shall appear to forestall the Committee's judgment, even in trivial things, supposing they can be expected to record one, it is not too early to enter a protest against the indulgence of a too obvious animus against a great institution, worthy, we hope, of its own traditions and not likely to suffer by bonâ fide inquiry and criticism.

If there be any hospital manager with mind narrow enough to find satisfaction in the indictment of the greatest of our metropolitan medical institutions, we would venture to remind him that the condemnation of "The London" would be a misfortune of national dimensions and the heaviest blow yet dealt at the voluntary system. Truly the hospitals are sorely tried when, in addition to the other ills they suffer, they find themselves at the mercy of any discharged servant or incompetent nurse who has a grudge against the powers which were. And, as has been justly observed, the public too often hear the complaint without troubling themselves about the reply.

Imagine the consequences of devoting the time of this august assembly to a discussion of the question whether nurses should trim lamps and fill inkstands, or whether the ward linen is ample! We have a right to ask respectfully whether it was for this a Select Committee was granted, and it is because we fear that the greater subjects which await their lordships' examination may suffer neglect that we venture to press the question.

Of course there is, as there always will be, room for improvement in a variety of directions, and the nurses, as a body, are no doubt worthy of more generous treatment, but at present it seems open to any person who has ever entered the door of a metropolitan hospital—one witness who presented himself had not even done that, we believe—to come forward and formulate statements for the value or accuracy of which there is no guarantee. It is obvious that if this facility exists, some out of the thousands of persons of all sorts who become the inmates of hospitals for longer or shorter periods, in various positions, will be found capable of an attempt to serve their own ends without a spark of interest in the subject as a whole. Let us be thankful all are not as the malcontents. It is refreshing to be relieved of their trivialities as we turn to the really interesting and valuable evidence of certain of the medical witnesses. Whether we agree or not with all they say, we cannot fail to recognise that we are reading the evidence of men whose capacity and bona fides are unimpeachable, and who can place something upon record which when it is debateable is at least worthy of debate. Can we say the same of those witnesses to whom a chance of publishing personal grievances may be the only inducement to give tongue?

